# Cutaneous Metastases from Primary Hepatobiliary Tumors as the First Sign of Tumor Recurrence following Liver Transplantation

**DOI:** 10.1155/2014/838949

**Published:** 2014-07-10

**Authors:** Adam T. Hauch, Joseph F. Buell, Margit McGowan, Parisha Bhatia, Eleanor Lewin, Mary Killackey, Nathan J. Shores, Luis A. Balart, Martin Moehlen, Bob Saggi, Anil S. Paramesh

**Affiliations:** ^1^Tulane Transplant Institute, Tulane University School of Medicine, 1415 Tulane Avenue, HC-5, New Orleans, LA 70112, USA; ^2^Department of Hematology and Oncology, Tulane University School of Medicine, New Orleans, LA 70112, USA; ^3^Department of Pathology, Tulane University School of Medicine, New Orleans, LA 70112, USA; ^4^Department of Gastroenterology, Section of Hepatology, Tulane University School of Medicine, New Orleans, LA 70112, USA

## Abstract

Cutaneous metastasis from hepatobiliary tumors is a rare event, especially following liver transplantation. We report our experience with two cases of cutaneous metastases from both hepatocellular carcinoma and mixed hepatocellular/cholangiocarcinoma following liver transplantation, along with a review of the literature.

## 1. Introduction

Cutaneous metastases from internal malignancies occur in 0.6%–10.4% of cancer patients and represent just 2% of all skin tumors [1, 2]. They often present with variable clinical appearance, which may result in a delay and failure in making their diagnosis, impacting morbidity, prognosis, and treatment [2]. Furthermore, they may represent the first sign of cancer recurrence and hence require a high index of suspicion for making the diagnosis.

Skin metastases from primary hepatobiliary malignancies, such as hepatocellular carcinoma (HCC) or cholangiocarcinoma, are exceedingly rare events described mostly in case reports [3–7]. It is estimated that cutaneous metastasis from HCC represents only 0.2%–2.7% of all cutaneous metastases [8], and metastatic lesions to the skin secondary to cholangiocarcinoma are even more infrequent with less than thirty documented reports in the literature [7].

Although rare, cutaneous metastases may represent the first sign of HCC or cholangiocarcinoma recurrence. To date, there has been only one documented case describing skin metastasis from HCC in a patient who underwent liver transplantation [9]. In this report, we will describe our experience with the diagnosis and management of cutaneous hepatobiliary metastases in two patients following liver transplantation.

## 2. Case  1

The patient is a 60-year-old Caucasian male who underwent liver transplantation on 1/24/2011 for cirrhosis secondary to Hepatitis C virus (HCV) infection, with a biopsy proven Stage II HCC (3.2 cm single lesion). He underwent chemoembolization prior to transplant. Surgical pathology of the native liver explant demonstrated a residual 2 cm well-differentiated HCC with no lymphovascular invasion and negative margins.

**Figure 1:**
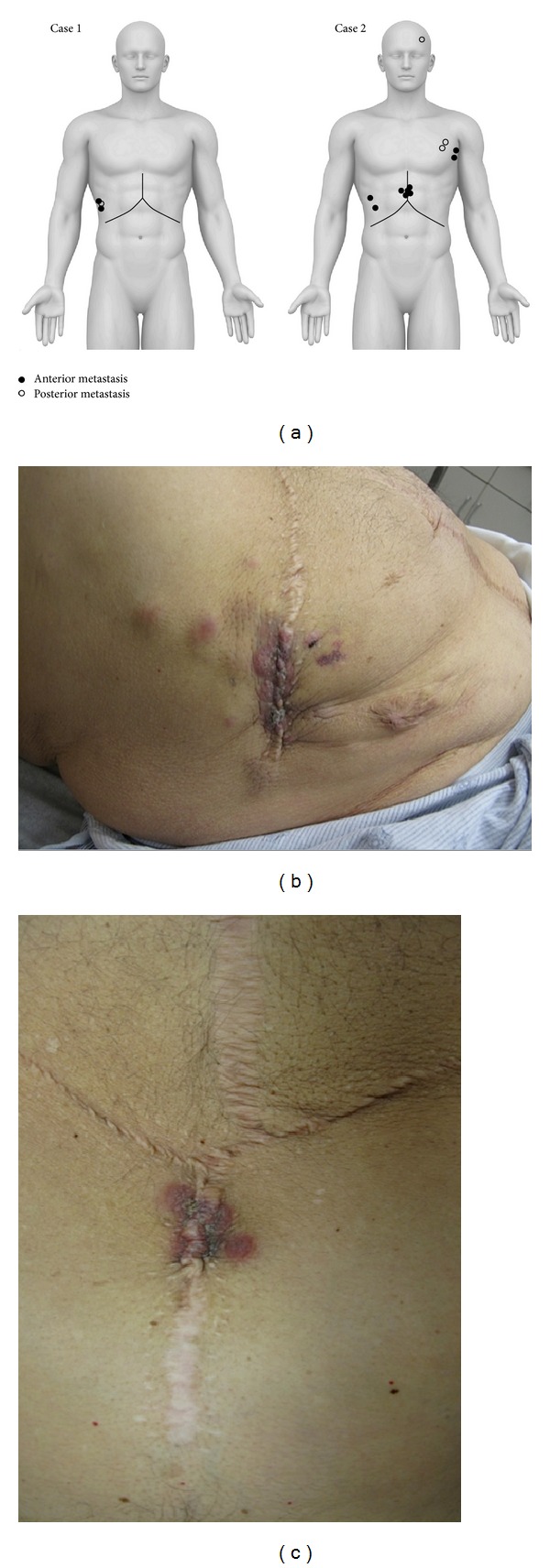
(a) Illustration demonstrating locations of cutaneous metastases for patients of Case  1 and Case  2. (b and c) Multiple erythematous-violaceous metastatic lesions with irregular borders representing mixed HCC/cholangiocarcinoma found at the previous incision site of the patient in Case  2.

**Figure 2:**
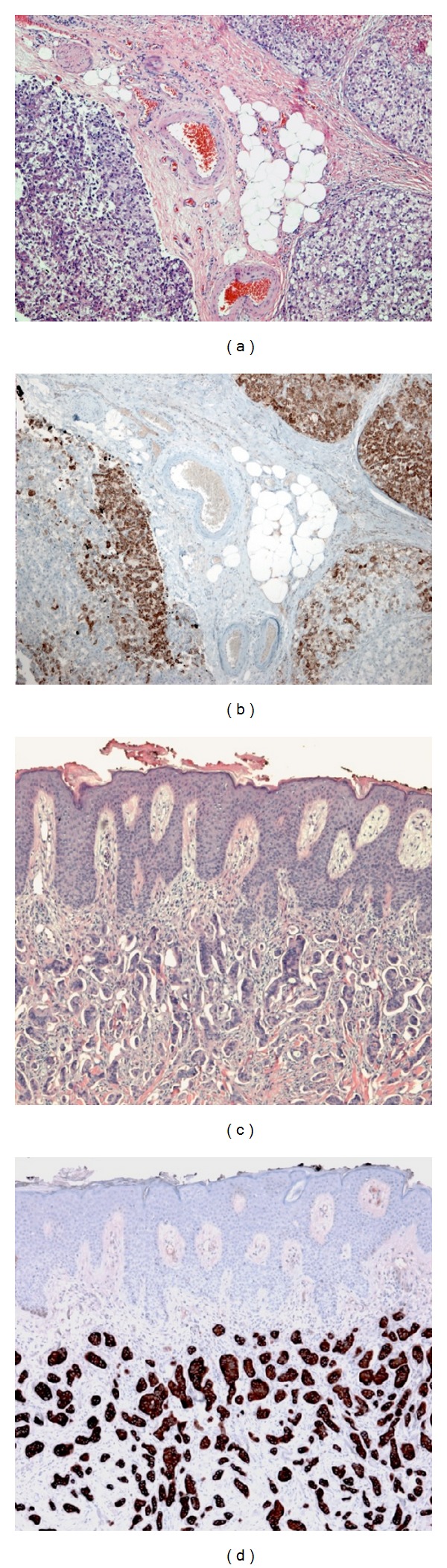
(a) H&E staining of abdominal wall excisional biopsy showing nodules of tumor cells with intervening entrapped soft tissue. Tumor cells demonstrate large variation in size with moderate nuclear pleomorphism; many tumor cells show cytoplasmic clearing. (b) Tumor cells show strong focal cytoplasmic reactivity for HepPar-1 supporting a diagnosis of metastatic HCC. (c) H&E staining of punch biopsy of skin showing tumor cells in the dermis streaming in cords and trabeculae with occasional gland-like formations. Tumor cells are hyperchromatic with increased nuclear-to-cytoplasmic ratio. (d) Tumor cells highlighted by CK7 immunohistochemistry (brown), showing diffuse strong cytoplasmic reactivity. Tumor cells were also positive for EMA and focally positive for mucicarmine; CK20, HepPar-1, CDX-2, and PSA were negative. The immunoprofile (CK7(+), CK20(−), and HepPar-1(−)) supports biliary over hepatic origin but is not definitive.

The patient initially had good liver function but developed recurrent HCV liver disease about one year after transplant and was initiated on antiviral therapy with an interferon, ribavirin, and boceprevir protocol. In December 2013 the patient developed several violaceous, painful, firm, immobile skin lesions on his right anterior chest wall, several inches from his transplant incision (Figure 1(a)). These lesions were biopsied and demonstrated subcutaneous metastatic well-differentiated HCC (Figure 2(a)). Immunohistochemical staining was positive for HepPar-1 (Figure 2(b)). Interestingly, imaging did not reveal any evidence of intrahepatic or pulmonary HCC recurrence. The patient had undergone a percutaneous biopsy of his HCC pretransplant, but this had been done in October of 2010, more than three years earlier. His alpha-fetoprotein (AFP) levels were never elevated.

With clinically and radiographically localized disease, the patient underwent metastectomy with wide local excision and mesh reconstruction in January 2014. Surgical pathology revealed three nodules, the largest measuring 3.7 cm, with each revealing a moderately differentiated HCC. Lymphovascular invasion was also present. After resection, the patient's immunosuppression was changed from a tacrolimus-based to an everolimus-based regimen, due to its potential antitumor properties, and sorafenib therapy was initiated.

## 3. Case  2

The second patient is a 55-year-old Caucasian male who underwent orthotopic liver transplantation on 4/7/2012 for HCV-associated cirrhosis and biopsy proven well-differentiated Stage II HCC (4.9 cm single lesion). He underwent chemoembolization of this mass prior to transplant. Posttransplant surgical pathology of the native liver explant revealed a minimal focus (0.5 cm) of residual HCC at the previous chemoembolization site, as well as another 2.5 cm adenocarcinoma in the perihilar region of the liver, which was suggestive by morphologic appearance and immunophenotypic profiling (positive for CK7, CK20, and mCEA) to be consistent with a mixed HCC/cholangiocarcinoma. This tumor demonstrated microscopic lymphatic, vascular, and perineural invasion with bile duct dysplasia. The lesion had not been seen on pretransplant imaging studies or during his hepatic angiogram during chemoembolization. Because of this, he was maintained with a low-dose immunosuppression regimen and closely monitored for recurrence.

In December 2013, eighteen months following transplant, the patient developed numerous painful skin nodules on his abdomen along the lateral and superior aspects of his previous incision site, chest wall, and scalp (Figure 1). A biopsy and histopathologic examination of one of the skin lesions revealed tumor cells which were positive for CK7 and EMA immunostains and which were morphologically compatible with metastatic cholangiocarcinoma/HCC (Figures 2(c) and 2(d)). Again, imaging did not reveal any evidence of intrahepatic tumor recurrence, or any other site of metastasis. His AFP levels remained normal as they had been before transplant, but his CA19-9 level in January 2014 was markedly elevated at 5600 U/mL (not done before transplant).

This patient's immunosuppression was also changed from a tacrolimus- to an everolimus-based regimen. He was treated with palliative electron beam radiation therapy to the skin lesions (250 cGy/d given as 14 fractions to 3500 cGy) and was initiated on a systemic chemotherapy regimen of gemcitabine (1000 mg/m^2^ IV on days 1 and 8) and cisplatin (25 mg/m^2^ IV on days 1 and 8) every 21 days with the goal of completing six cycles in total. In March 2014, he developed a large pleural effusion, which thoracentesis confirmed as malignant. Due to a rapidly deteriorating performance status, chemotherapy was discontinued and hospice services were initiated.

## 4. Discussion

The incidence of hepatocellular carcinoma is increasing in the United States and Europe, and it is the third highest cause of cancer-related death globally [10]. HCC is one of the few indications of a tumor that may be potentially cured with liver transplantation, by elimination of both the tumor and the preneoplastic background of underlying liver cirrhosis.

Patients within the Milan criteria (defined as no gross vascular invasion with either a single tumor ≤5 cm or three tumors with the largest lesion <3 cm; up to Stage II tumors) are given priority on transplant waiting lists following workup to rule out metastases [11]. Although recurrence of HCC following liver transplantation has significantly declined since the adoption of the Milan criteria, 8–12% of patients will still develop recurrence with the majority of these patients developing advanced forms of multifocal and extrahepatic metastases. The most common metastatic sites are lung, bones, lymph node, and adrenal glands; cutaneous metastases occur much less frequently [4].

Cutaneous metastases from HCC are estimated to represent only 0.2%–2.7% of all cutaneous metastases [8]. Cholangiocarcinoma tends to spread to adjacent organs with less than thirty cases of cutaneous metastases described in the literature [7].

Metastasis to the skin may occur by several different pathways, including hematogenous and lymphatic spread, direct contiguous tissue invasion, and iatrogenic implantation [8]. Cutaneous metastases from HCC most commonly occur in the face, scalp, chest, and shoulders and appear as single or multiple 1–5 cm lesions, which are often firm and reddish blue. Ulceration is often absent and rapid growth is common [4, 8]. These skin lesions may resemble pyogenic granulomas [5] or subcutaneous abscesses [4] or have a hemangiomatous morphology with profuse bleeding due to tumor hypervascularity [6]. Cutaneous metastases from cholangiocarcinoma may also occur in the scalp and face, but they are more often located on the abdominal wall (i.e., at the exit-site of a percutaneous biliary drain) [8].

In our first case, the etiology of the cutaneous metastases is unclear. The original tumor did not appear to have any lymphatic invasion although this may be due to the ablative chemoembolization. The patient had a CT guided biopsy at an outside institution, and it is possible that the site of metastases may have been the site of needle insertion. Still, this biopsy occurred three years earlier making the diagnosis of needle tracking less likely. There was no tumor spillage in either of the cases.

Histopathology of cutaneous HCC demonstrates a bottom heavy architecture, with the main infiltrate located in the deep dermis, and the presence of trabecular and pseudoglandular patterns composed of acidophilic cells [3, 8]. Tumor necrosis and vascular invasion are common findings. Furthermore, Mallory bodies (intracytoplasmic inclusion bodies found in hepatocytes) strongly suggest HCC and the presence of bile is confirmative [3, 8]. Cutaneous metastasis from cholangiocarcinoma presents with a nonspecific pattern of diffuse involvement of the dermis by pleomorphic epithelial cells with poorly preserved glandular architecture [8].

Immunohistochemical studies have added great value to the establishment of a pathologic diagnosis for these diseases. Neoplastic hepatocyte cells express immunoreactivity for polyclonal carcinoembryonic antigen (CEA), AFP, cytokeratins (CK) 8 and 18, and hepatocyte paraffin-1 (HepPar-1) (an antigen of the hepatocyte mitochondria) monoclonal antibody, which has been considered as the most specific and sensitive marker of normal and neoplastic hepatocytes [3, 8]. Although there is no characteristic panel of antibodies that are pathognomonic for cholangiocarcinoma, tumors of biliary epithelial origin tend to express CK7 and sometimes CK20 although they lack CDX2 expression [7, 8]. These immunohistochemical markers can thus be helpful in initially narrowing the diagnosis.

To date, there have been no reports of metastatic cholangiocarcinoma to the skin and only one case of metastatic HCC to the skin following liver transplantation [9]. In the report by Alonso-González, HCC recurrence was identified within the liver, lungs, and lymph nodes. The patient died just 6 weeks following their diagnosis of skin metastases. We present two cases of cutaneous metastases from hepatobiliary primaries, representing both HCC and mixed HCC/cholangiocarcinoma, as the first sign of tumor recurrence in two liver transplant recipients. Intrahepatic recurrence or metastases elsewhere were not identified in either case profile.

Skin metastases from liver cancers represent a dismal prognosis for most patients, with overall survival varying between a few weeks and six months [4, 9]. The patient who underwent abdominal wall resection and mesh reconstruction is doing well at the time of the publication of this paper, six months since diagnosis, while the other patient suffered a precipitous decline in his health with malignant pleural fluid and poorly controlled pain and died just three months following diagnosis. The first patient has been started on sorafenib, an oral multikinase inhibitor of the vascular endothelial growth factor receptor, the platelet-derived growth factor receptor *β*, and Raf, which has been shown to inhibit tumor-cell proliferation and angiogenesis and increase the rate of tumor-cell apoptosis in advanced cases of hepatocellular carcinoma and other tumors [12]. Two large randomized control trials have demonstrated that it can increase survival of patients with advanced HCC by approximately 2 to 3 months, and the Food and Drug Administration approved it in 2007 for this patient population [12, 13]. Recent case-control studies have demonstrated similar modest survival benefit in patients with recurrent HCC following liver transplantation [14, 15], and based on current data, algorithms that integrate sorafenib into the management of posttransplant HCC recurrence are emerging [16].

Additionally, both patients underwent modifications in immunosuppression and have been switched to everolimus due to the potential antitumor activities of this drug through its ability to inhibit the mTOR pathway, a key regulator of cellular proliferation and angiogenesis, the activation of which has been linked to a variety of malignancies [17]. Despite the potentially beneficial effects of mTOR inhibitors on HCC recurrence following liver transplantation, there are currently only retrospective and uncontrolled clinical trials available from which to draw supportive evidence [17]. Fortunately, a prospective, randomized open-label trial examining the application of mTOR inhibitors for the prevention of HCC recurrence following liver transplantation is currently underway [18].

In summary, the present report demonstrates that cutaneous metastases from hepatobiliary cancers may occur following liver transplantation. This may be the first sign of recurrence and a high degree of suspicion is warranted. Although skin metastases generally portend a grim prognosis, palliative options may include surgery, radiotherapy, immunosuppressant adjustments, and systemic chemotherapy.

## References

[1] Lookingbill DP, Spangler N, Helm KF (1993). Cutaneous metastases in patients with metastatic carcinoma: a retrospective study of 4020 patients. *Journal of the American Academy of Dermatology*.

[2] Nashan D, Meiss F, Braun-Falco M, Reichenberger S (2010). Cutaneous metastases from internal malignancies. *Dermatologic Therapy*.

[3] Terada T, Maruo H (2013). Unusual extrahepatic metastatic sites from hepatocellular carcinoma. *International Journal of Clinical and Experimental Pathology*.

[4] Amador A, Monforte NG, Bejarano N (2007). Cutaneous metastasis from hepatocellular carcinoma as the first clinical sign. *Journal of Hepato-Biliary-Pancreatic Surgery*.

[5] Kubota Y, Koga T, Nakayama J (1999). Cutaneous metastasis from hepatocellular carcinoma resembling pyogenic granuloma. *Clinical and Experimental Dermatology*.

[6] Ackerman D, Barr RJ, Elias AN (2001). Cutaneous metastases from hepatocellular carcinoma. *International Journal of Dermatology*.

[7] West KL, Selim MA, Puri PK (2010). Cutaneous metastatic cholangiocarcinoma: a report of three cases and review of the literature. *Journal of Cutaneous Pathology*.

[8] Alcaraz I, Cerroni L, Rütten A, Kutzner H, Requena L (2012). Cutaneous metastases from internal malignancies: a clinicopathologic and immunohistochemical review. *The American Journal of Dermatopathology*.

[9] Alonso-González J, Sánchez-Aguilar D, Toribio J (2012). Multiple cutaneous metastases from hepatocellular carcinoma as the first sign of tumor recurrence in a transplant patient. *Actas Dermo-Sifiliograficas*.

[10] Parkin DM, Bray F, Ferlay J, Pisani P (2005). Global cancer statistics, 2002. *Cancer Journal for Clinicians*.

[11] Mazzaferro V, Regalia E, Doci R (1996). Liver transplantation for the treatment of small hepatocellular carcinomas in patients with cirrhosis. *The New England Journal of Medicine*.

[12] Llovet JM, Ricci S, Mazzaferro V (2008). Sorafenib in advanced hepatocellular carcinoma. *New England Journal of Medicine*.

[13] Cheng AL, Kang YK, Chen Z (2009). Efficacy and safety of sorafenib in patients in the Asia-Pacific region with advanced hepatocellular carcinoma: a phase III randomised, double-blind, placebo-controlled trial. *The Lancet Oncology*.

[14] Waghray A, Balci B, El-Gazzaz G (2013). Safety and efficacy of sorafenib for the treatment of recurrent hepatocellular carcinoma after liver transplantation. *Clinical Transplantation*.

[15] Sposito C, Mariani L, Germini A (2013). Comparative efficacy of sorafenib versus best supportive care in recurrent hepatocellular carcinoma after liver transplantation: a case-control study. *Journal of Hepatology*.

[16] Toso C, Mentha G, Majno P (2013). Integrating sorafenib into an algorithm for the management of post-transplant hepatocellular carcinoma recurrence. *Journal of Hepatology*.

[17] Chen K, Man K, Metselaar HJ, Janssen HL, Peppelenbosch MP, Pan Q (2014). Rationale of personalized immunosuppressive medication for hepatocellular carcinoma patients after liver transplantation. *Liver Transplantation*.

[18] Schnitzbauer AA, Zuelke C, Graeb C (2010). A prospective randomised, open-labeled, trial comparing sirolimus-containing versus mTOR-inhibitor-free immunosuppression in patients undergoing liver transplantation for hepatocellular carcinoma. *BMC Cancer*.

